# Diagnostic pitfalls: soft-tissue sarcomas initially misdiagnosed as benign vascular anomalies—a case report and systematic review

**DOI:** 10.3389/or.2025.1681090

**Published:** 2025-12-11

**Authors:** Malgorzata Styczewska, Weronika Lyzinska, Stanislaw Maria Wardecki, Joanna Zajaczkowska, Michal Kunc, Boguslaw Mikaszewski, Rafal Maciag, Bartosz Regent, Dariusz Wyrzykowski, Anna Jankowska, Dominik Swieton, Katarzyna Sinacka, Katarzyna Zak-Jasinska, Anna Jedrzejczyk, Malgorzata A. Krawczyk, Ewa Bien

**Affiliations:** 1 Department of Pediatrics, Hematology and Oncology, Medical University of Gdansk, Gdansk, Poland; 2 Department of Pediatrics, Hematology and Oncology, University Clinical Center in Gdansk, Gdansk, Poland; 3 The English Division Pediatric Oncology Scientific Circle, Medical University of Gdansk, Gdansk, Poland; 4 Department of Pathology, Medical University of Gdansk, Gdansk, Poland; 5 Department of Otolaryngology, Medical University of Gdansk, Gdansk, Poland; 6 2nd Department of Clinical Radiology, Medical University of Warsaw, Warsaw, Poland; 7 Department of Radiology, Medical University of Gdansk, Gdansk, Poland; 8 Department of Pediatric Surgery and Urology, Copernicus Hospital in Gdansk, Gdansk, Poland; 9 St. Lawrence Hospice Association, Gdynia, Poland

**Keywords:** vascular malformation, sarcoma, hemangioma, vascular anomalies, misdiagnosis

## Abstract

**Introduction:**

Vascular anomalies (VAs), comprising vascular tumors and malformations, are commonly diagnosed based solely on clinical evaluation and imaging. Soft-tissue sarcomas (STSs) may mimic VAs clinically and radiologically, leading to misdiagnosis, delayed treatment, and suboptimal outcomes. In this systematic review, we aimed to summarize patients with a pathological diagnosis of STSs who were initially misdiagnosed with benign VAs, highlighting diagnostic pitfalls.

**Materials and methods:**

In this systematic review (PROSPERO ID: CRD42024615285), we followed the PRISMA 2020 guidelines. The inclusion criteria comprised patients with histologically confirmed STSs who had been initially misdiagnosed as benign VAs based on clinical or radiological features. Literature from five databases was reviewed without language or date restrictions. One additional case of alveolar soft-part sarcoma initially misdiagnosed and mistreated as an arteriovenous malformation from the authors’ institution was added to the analysis.

**Results:**

The systematic search yielded a total of 96 patients with STS initially misdiagnosed as benign VAs (95 from 77 publications and one from our own case). The median age at presentation was 6 months (range: newborn–88 years). The most frequent symptom was a swelling or mass (75%). In most cases, the misdiagnosis was both clinical and radiological. The median diagnostic delay was 5.5 months. Fifty-nine (61.5%) patients received treatment for the misdiagnosed benign VA, including local interventions (51.0%) and systemic therapies (17.7%). The most commonly misdiagnosed STS subtypes were infantile fibrosarcoma, alveolar soft-part sarcoma, rhabdomyosarcoma, dermatofibrosarcoma protuberans, angiosarcoma, and Ewing sarcoma.

**Conclusion:**

Several STS subtypes may mimic benign VAs clinically and radiologically. The misuse of outdated terminology and limited awareness among clinicians contribute to diagnostic delays. To avoid misdiagnoses, the care for patients with benign VAs should be provided by specialists familiar with the classification and natural history of these lesions. In patients diagnosed with benign VAs based on clinical or imaging features only, all findings should clearly support the diagnosis. Any ambiguity warrants prompt referral to a tertiary center. A biopsy should be considered in doubtful or atypical cases.

## 1 Introduction

Vascular anomalies (VAs) are disorders characterized by abnormal development or growth of blood or lymphatic vessels. According to the International Society for the Study of Vascular Anomalies (ISSVA) classification, updated in 2025, VAs can be divided into two groups: vascular tumors—neoplasms associated with excessive endothelial cell proliferation—and vascular malformations, which are congenital, structural abnormalities of blood or lymphatic vessels, without considerable proliferative activity (1). Vascular anomalies are primarily diagnosed based on clinical evaluation, often supported by imaging techniques such as Doppler ultrasound and magnetic resonance imaging (MRI) (2). Given the risk of bleeding and potential complications, a biopsy is usually reserved for cases of diagnostic uncertainty (2,3). However, the differentiation between VAs and other pathological entities can be challenging, sometimes leading to misdiagnosis and inappropriate management.

Soft-tissue sarcomas (STSs) are a heterogeneous group of malignant tumors arising from mesenchymal tissues, capable of developing in various anatomical locations. Despite their low incidence—approximately 5 cases per 100,000 individuals (4)—they account for 1% of all solid malignancies in adults and up to 21% of malignant tumors in children (5). Diagnosing STSs is particularly challenging due to their rarity, the absence of a specific clinical presentation, and their substantial radiological heterogeneity, reflecting the existence of over 70 histological subtypes of STSs (4,5).

Notably, STSs can mimic both benign and malignant VAs, leading to potential misdiagnosis when relying solely on clinical and radiological findings. This diagnostic challenge can result in delayed or inappropriate treatment (6), ultimately affecting patient outcomes (7). This systematic review aims to analyze reported cases in which STSs were initially misdiagnosed as benign VAs, highlighting the clinical and imaging pitfalls that contribute to these mistakes and emphasizing the importance of a thorough diagnostic approach.

## 2 Materials and methods

**FIGURE 1:**
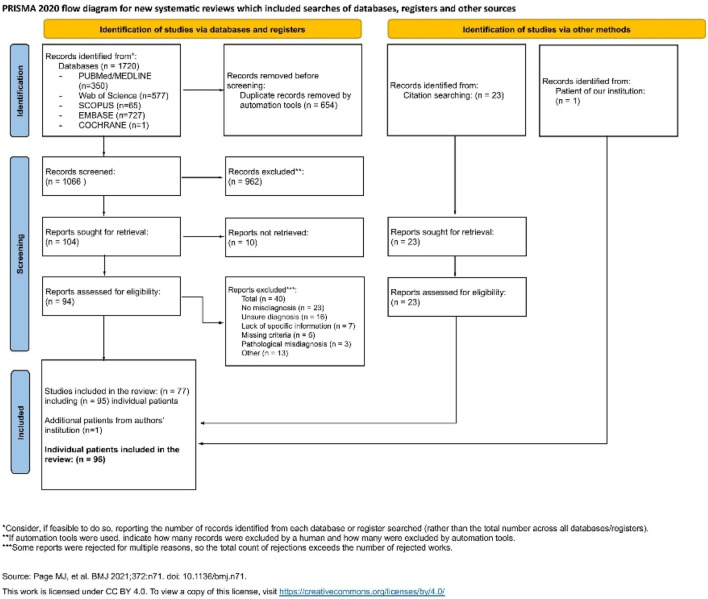
A PRISMA flowchart of the systematic review.

The systematic review has been registered in the PROSPERO database (ID: CRD42024615285) and conducted according to the Preferred Reporting Items for Systematic Reviews and Meta-Analyses (PRISMA) 2020 guidelines [Figure 1]. The Population, Intervention, Comparison, Outcomes, and Study Design (PICOS) for the study are described in Table 1. The inclusion criteria were as follows: patients with a histopathological diagnosis of STS who had been initially misdiagnosed with benign VAs based on clinical or radiological features. Soft-tissue sarcomas have been defined as malignant and borderline/locally aggressive soft-tissue neoplasms, according to the World Health Organization (WHO) Classification of Tumours, 5th edition (8), with the exclusion of borderline vascular tumors. Only soft-tissue tumors were included (we excluded STS primarily involving internal organs, e.g., liver, spleen, or lungs). Benign VAs have been defined as benign vascular tumors and vascular malformations according to the 2018 ISSVA Classification (9). There was no restriction on the language or year of publication.

**TABLE 1:** Demographic and clinical data of analyzed patients.

All patients		*n* = 96 (100%)
Own case		1 (1.0%)
Literature reports		95 (99.0%)
Sex, n (%)		
Female		39 (40.6)
Male		45 (46.9)
Unknown		12 (12.5)
Median age at presentation [years]		0.5 (range: 0–88, IQR 0–10.25)
Tumor site, n (%)		
Extremities		50 (52.1)
Head and neck		31 (32.3)
Trunk		12 (12.5)
Head and neck + trunk		1 (1.0)
Multifocal		2 (2.1)
Median tumor size at presentation [cm]		4 (range: 0.3–10, IQR 3–6)
Symptom^a^		
Swelling/mass		72 (75.0)
Pain		9 (9.4)
Bleeding or anemia		6 (6.3)
Ocular symptoms		6 (6.3)
Plaque		4 (4.2)
Ulceration		4 (4.2)
Papule		3 (3.1)
Visible collateral vessels		2 (2.1)
Speech and swallowing difficulties		2 (2.1)
Discomfort		1 (1.0)
Cholestasis		1 (1.0)
Initial misdiagnosis^b^		
Hemangioma, n = 57 (59.4%)	Not specified	36 (37.5)
	Congenital hemangioma	9 (9.4)
	Infantile hemangioma	4 (4.2)
	Capillary hemangioma	2 (2.1)
	Lobular capillary hemangioma	2 (2.1)
	Cavernous hemangioma	1 (1.0)
	Tufted hemangioma	1 (1.0)
	Atypical hemangioma	1 (1.0)
	Disseminated hemangiomatosis	1 (1.0)
Vascular malformation, n = 32 (33.3%)	Lymphatic/lymphangioma/cystic hygroma	12 (12.5)
	AVM/high flow	10 (10.4)
	Not specified	9 (9.4)
	Lympho-venous	1 (1.0)
Multiple benign VAs n = 7 (7.3%)	-	7 (7.3)
Type of misdiagnosis, n (%)		
Clinical		38 (39.6)
Radiological		7 (7.3)
Both clinical and radiological		44 (45.8)
Not provided		7 (7.3)
Median diagnostic delay [months]		5 (range 0.25–360, IQR 2–23.25)
Treatment of the misdiagnosed VAs^c^		
Local, n = 49 (51.0%)	Surgery	42 (43.8)
	Embolization/chemoembolization	9 (9.4)
	Laser therapy	5 (5.2)
	Sclerotherapy	1 (1.0)
	Cryosurgery	1 (1.0)
	Intralesional GCS	2 (2.1)
	Topical timolol	1 (1.0)
	Topical thrombin	1 (1.0)
Systemic, n = 17 (17.7%)	GCS	13 (13.5)
	Propranolol	7 (7.3)
	CHT	2 (2.1)
	Sirolimus	2 (2.1)
	Antibiotics	2 (2.1)
	Interferon alpha-2a	1 (1.0)
Final diagnosis of STS, n (%)		
Infantile fibrosarcoma		23 (24.0)
Alveolar soft-part sarcoma		23 (24.0)
Rhabdomyosarcoma		13 (13.5)
Dermatofibrosarcoma protuberans		8 (8.3)
Angiosarcoma		6 (6.3)
Ewing sarcoma		5 (5.2)
Kaposi sarcoma		4 (4.2)
Other		14 (14.6)
Outcome, n (%)		
No evidence of disease (NED)		52 (54.2)
Died of disease (DOD)		15 (15.6)
Alive with disease (AWD)		13 (13.5)
Not specified		16 (16.7)
Median follow-up [months]		16.5 (range: 0–144; IQR: 7.75–29)

AVM, arteriovenous malformation; CHT, chemotherapy; cm, centimeters; GCS, glucocorticoids; STS, soft-tissue sarcoma; VAs, vascular anomalies.

^a^ Percentages do not sum up to 100% as several patients experienced multiple symptoms.

^b^ Original names used in the publications have been retained.

^c^ Percentages do not sum up to 100% as several patients received multiple treatment methods.

Publications listed in the PubMed/MEDLINE, Embase, Cochrane Central Register of Controlled Trials (CENTRAL), Scopus, and Web of Science databases up to 17.11.2024 have been searched. The screening (database search, article retrieval, and data analysis) was performed by four independent reviewers (M.S., W.L., J.Z., and S.W.). Duplicated publications were removed using an automatic tool [Rayyan (10)] and manually. The search was performed using both subject headings (MeSH and Emtree terms in MEDLINE and Embase, respectively) and free-text keywords. The following keyword search terms were included: “sarcoma,” “rhabdomyosarcoma,” “leiomyosarcoma,” “angiosarcoma,” “liposarcoma,” “dermatofibrosarcoma” or “fibrosarcoma” combined with “vascular malformation,” “arteriovenous malformation,” “venous malformation,” “lymphatic malformation,” “capillary malformation,” “hemangioma” or “vascular anomaly” combined with “misdiagnosis,” “misdiagnosed,” “mistake,” “mistaken,” “pitfall,” “differential,” “mimicking,” or “mimicker.” A full search protocol has been provided in Supplementary Material S1. No additional search filters have been applied. The reference lists in all publications have also been searched for additional cases.

The data retrieved from each article included the following: the age and sex of the patient, information on the symptoms at presentation, the site and size of the lesion, type of benign VA that was misdiagnosed, diagnostic delay, type of misdiagnosis (clinical or radiological), final diagnosis of STS, treatment before and after the proper diagnosis of STS, follow-up, and outcome. The demographic data from the patients have been carefully analyzed to avoid duplication of cases.

The first presentation was defined as the time of the first symptoms of the disease, regardless of whether the patient contacted healthcare professional or underwent diagnostic procedures. The diagnostic delay was defined as the time between the first presentation and the histopathological diagnosis of STS. The follow-up was defined as the time between the histopathological diagnosis of STS and death or the last contact with the patient. In cases with an uncertain duration of diagnostic delay or follow-up, we reported the longest precisely reported timespan. Original denominations of the misdiagnosed benign VAs used in the cited publications have been retained, even if incorrect according to the current ISSVA Classification.

The series from the literature was completed by one case of our own, involving a 16-year-old patient with alveolar soft-part sarcoma (ASPS) initially misdiagnosed as arteriovenous malformation (AVM). A written informed consent for the publication of the case report and photographs was obtained from the parents.

## 3 Case report

**FIGURE 2:**
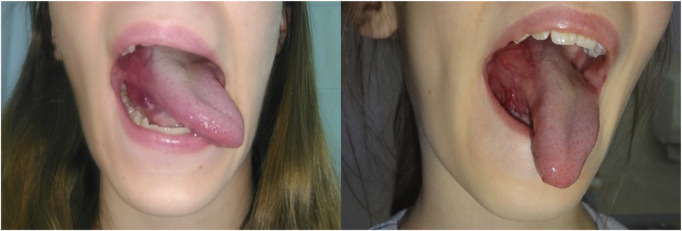
Initial clinical presentation—a highly vascularized lesion involving the base of the tongue on the right side.

**FIGURE 3:**
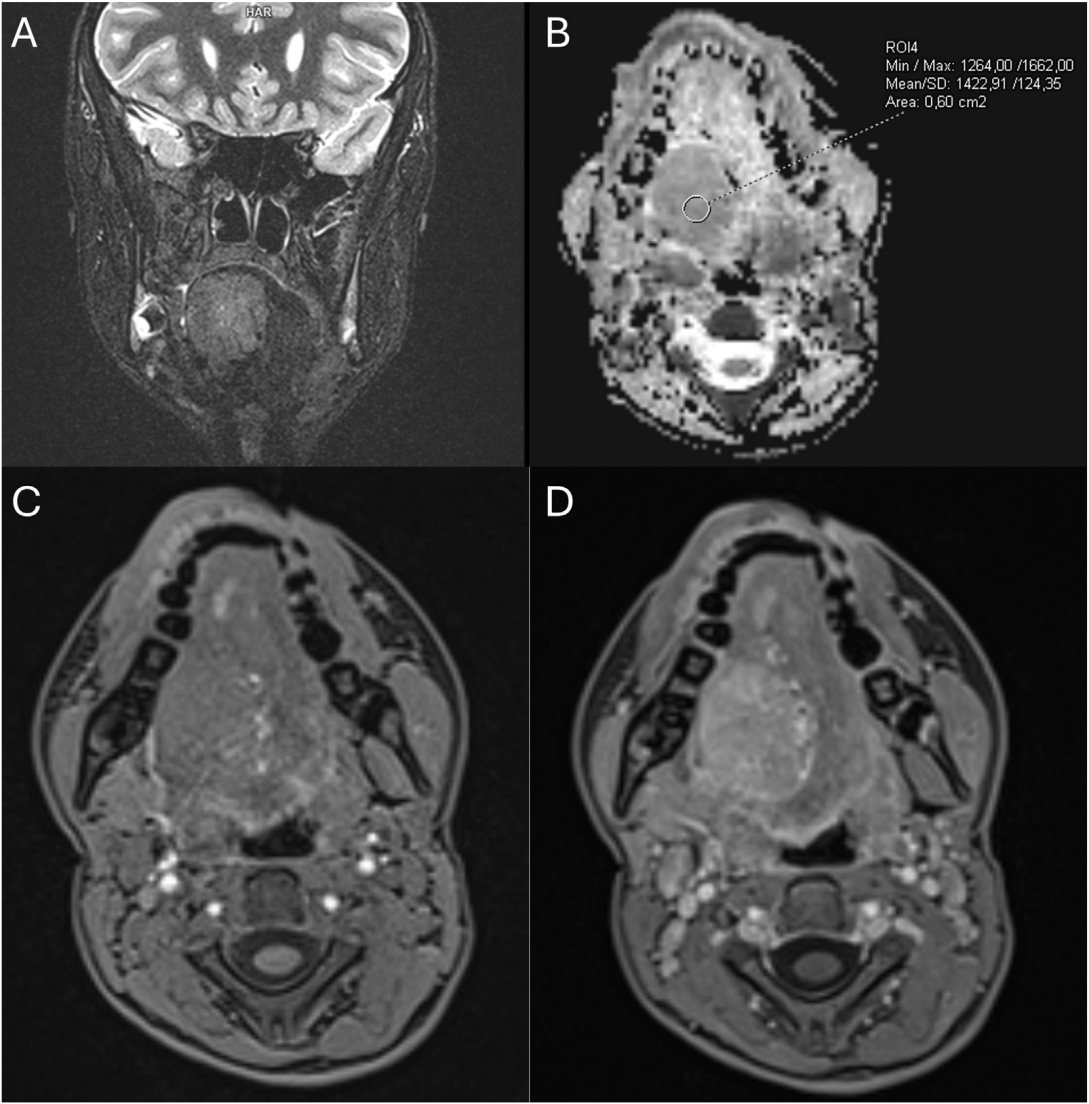
The first MRI examination in December 2021. **(A)** Coronal T2-weighted MRI image with fat saturation showing a pathologic mass in the tongue. **(B)** MRI-apparent diffusion coefficient (ADC) map highlighting the tongue mass. **(C,D)** Pre- and post-contrast T1-weighted gradient-echo MRI images demonstrating vivid enhancement of the tongue mass and dilated surrounding vessels.

A 10-year-old female patient presented in 2017 with a lesion involving the base of the tongue, which was diagnosed as an AVM based on clinical assessment and Doppler ultrasound findings (Figure 2). At that time, no treatment was deemed necessary, and the patient remained under surveillance in the otolaryngology and pediatric surgery outpatient departments. In 2021, a significant progression of the disease occurred. Repeated radiological examinations (Doppler ultrasound and MRI) were, again, suggestive of AVM (Figure 3), and the rapid progression was attributed to the well-known phenomenon of accelerated growth of AVM during puberty (11). A biopsy was not performed due to the high risk of bleeding. Since 2022, the patient had been treated with ethanol and n-butyl cyanoacrylate (OnyxTM) embolizations, initially with good results. However, the first procedure was complicated with a 2-week-long intensive care unit (ICU) hospitalization, pneumonia, and respiratory distress requiring tracheostomy. In March 2023, consulting pediatric oncologists recommended a biopsy to obtain material for the molecular examinations, which could potentially enable qualification of the patient for systemic medical therapy. Nevertheless, following surgical consultation, the procedure was deemed too risky, and ultimately, it was not performed. Over the subsequent months, the embolization procedures became less effective. The patient experienced recurrent massive bleedings from the lesion and increasing difficulties with swallowing and speech. Therefore, in December 2023, she was admitted to the pediatric oncology department for evaluation and potential initiation of the systemic therapy.

**FIGURE 4:**
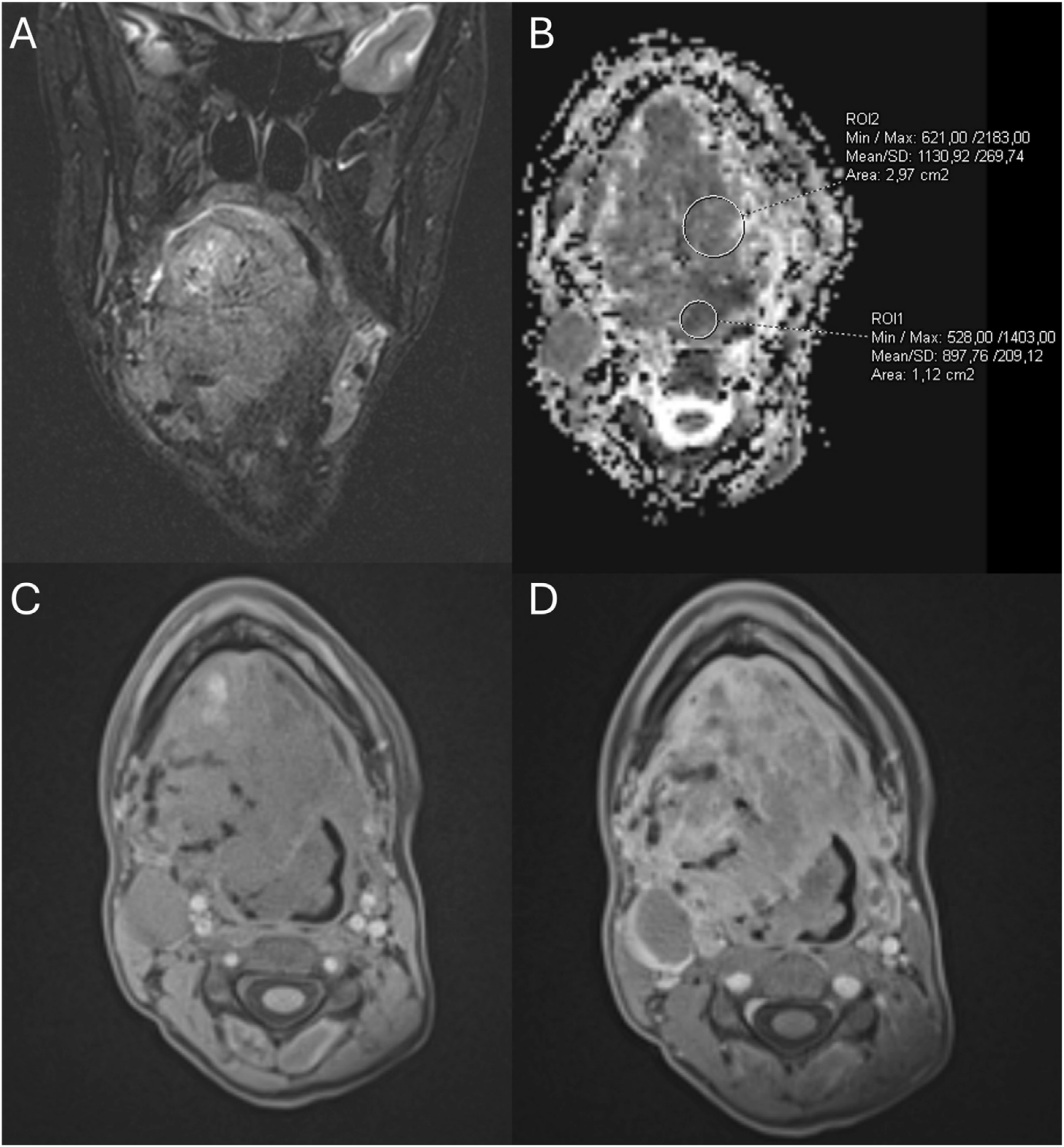
MRI examination at diagnosis of ASPS in December 2023. **(A)** Coronal T2-weighted MRI with fat saturation revealing a pathological mass in the tongue. **(B)** MRI-apparent diffusion coefficient (ADC) map displaying the pathological mass in the tongue. **(C,D)** Pre- and post-contrast T1-weighted gradient-echo MRI images showing vivid enhancement of the tongue mass and dilated surrounding vessels.

**FIGURE 5:**
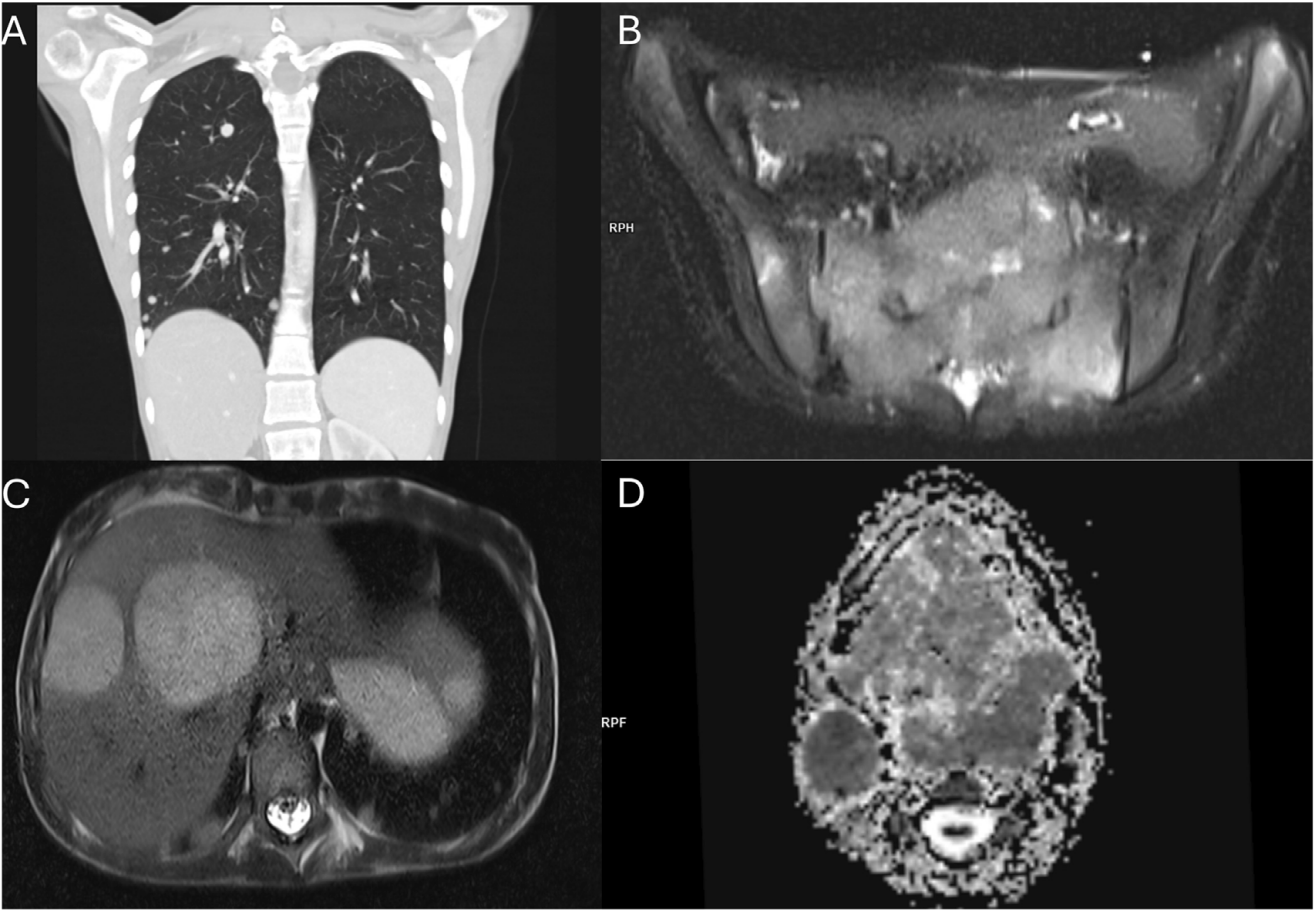
Staging imaging examinations at diagnosis of ASPS in 12.2023. **(A)** Coronal reconstruction of the lung CT scan demonstrating multiple metastatic lesions in the lung parenchyma. **(B)** T2-weighted MRI with fat saturation showing a sacral bone metastasis. **(C)** T2-weighted MRI revealing metastatic lesions in the liver. **(D)** MRI-apparent diffusion coefficient (ADC) map depicting the tongue mass and pathological cervical lymph nodes.

Upon admission, the patient was severely cachectic despite receiving enteral nutrition via gastrostomy for the past few months (BMI 13.9 kg/m^2^). Laboratory studies revealed markedly elevated inflammatory markers (CRP 180 mg/l and ESR 78 mm/h). MRI demonstrated an extensive pathological head and neck mass, measuring approximately 250 cm^3^, involving 90% of the tongue, along with the oral and laryngeal pharynx, oral cavity floor, epiglottis, submandibular and submental spaces, and right infratemporal fossa, raising suspicion of malignancy (Figure 4). Further imaging examinations revealed pathological cervical lymph nodes measuring up to 20 × 22 mm and numerous metastases to the lungs, bones, and the liver, consistent with disseminated neoplastic disease (Figure 5). Histopathological examination of the pathological cervical lymph node and lung nodules revealed ASPS, with the identification of the characteristic ASPSCR1:TFE3 fusion. The patient was treated with immunotherapy (atezolizumab) and targeted therapy (sunitinib) following the recommendations for ASPS, with no clinical response. After 3.5 months from the diagnosis of ASPS, the patient died due to the disease progression.

## 4 Systematic review

The systematic literature search identified 95 patients with STS misdiagnosed as benign VAs, reported in 77 publications. One additional case from our institution (detailed above) was added to the final analysis, resulting in a cohort of 96 patients (Figure 1; Table 2, Supplementary Material S2).

**TABLE 2:** PICOS framework.

PICOS framework	
Population	Patients of all ages with histopathologically confirmed soft-tissue sarcomas (STSs) who were initially misdiagnosed with benign vascular anomalies (VAs) based on clinical or radiological evaluation. The population includes a systematic review of 95 patients from the published literature and one new case from the authors’ institution
Intervention/exposure	Initial clinical or radiological misdiagnosis of benign VA, leading to delayed diagnosis of STS and/or incorrect treatment (e.g., embolization, propranolol, and surgery) for a presumed benign condition
Comparison	No direct comparison group. However, implicit comparison is between initial misdiagnosis versus correct histopathological diagnosis and its timing
Outcome	➢ diagnostic delay (median 5.5 months, range: 1 week–30 years) ➢ incorrect treatment (51% local, 17.7% systemic therapies before proper diagnosis) ➢ prognosis after correct diagnosis (52 NED, 13 AWD, and 15 DOD among 80 patients with known outcomes) ➢ highlighting key misdiagnosis patterns and diagnostic pitfalls
Study design	Systematic review of published case reports and case series + individual case report from the authors’ center. Conducted per PRISMA 2020 guidelines, registered in PROSPERO (ID: CRD42024615285)

The median age of the patients at initial presentation was 6 months (range: newborn to 88 years, IQR: 0–10.25 years), with almost equal gender distribution (male-to-female ratio: 1.15:1). Lesions were most commonly located on the extremities (n = 50, 52.1%), followed by the head and neck region (n = 31, 32.3%); the trunk region (n = 12, 12.5%); the head, neck, and trunk region (n = 1, 1.0%); and multifocal presentation (n = 2, 2.1%). Clinical symptoms were reported in 84 patients (87.5%). The most common presentation was a swelling or palpable mass (n = 72, 75.0%), followed by plaque (n = 4, 4.2%) and papule (n = 3, 3.1%). Additionally, patients experienced pain (n = 9, 9.4%); bleeding or anemia (n = 6, 6.3%); ocular symptoms such as proptosis, strabismus, and restricted eyeball mobility (n = 6, 6.3%); ulceration (n = 4, 4.2%); visible collateral vessels (n = 2, 2.1%); speech and swallowing difficulties (n = 2, 2.1%); discomfort (n = 1, 1.0%); and cholestasis (n = 1, 1.0%). Several patients exhibited more than one symptom. The maximal dimension of the lesion at the time of first presentation was reported in 32 patients, with a median of 4 cm (range: 0.3–10 cm, IQR: 3–6 cm).

The initial misdiagnoses of benign VAs included: hemangioma in 57 (59.4%) patients; in some cases further specified as congenital hemangioma (n = 9, 9.4%), infantile hemangioma (n = 4, 4.2%), lobular capillary hemangioma/pyogenic granuloma (n = 2, 2.1%), “capillary hemangioma” (n = 2, 2.1%), and “atypical,” “cavernous,” and “tufted hemangioma” (n = 1, 1.0% each). Vascular malformations were initially diagnosed in 32 (33.3%) patients, further categorized as lymphatic (n = 12, 12.5%), arteriovenous/high-flow (n = 10, 10.4%), and lympho-venous (n = 1, 1.0%). In seven patients, multiple benign VAs were considered in the differential diagnosis. The type of misdiagnosis was specified in 89 patients and described as clinical only (n = 38, 39.6%), radiological only (n = 7, 7.3%), and both clinical and radiological (n = 44, 45.8%). The duration of diagnostic delay was known in 70 patients and ranged from 1 week to 30 years (median: 5.5 months, IQR: 2–23.25 months). Fifty-nine patients received treatment for the misdiagnosed benign VA, which included local interventions (n = 49, 51.0%) and systemic therapies (n = 17, 17.7%), with several patients receiving multiple lines of treatment. The final histopathological diagnoses included infantile fibrosarcoma (IFS; n = 23, 24.0%), ASPS (n = 23, 24.0%), rhabdomyosarcoma (RMS; n = 13, 13.5%), dermatofibrosarcoma protuberans (DFSP; n = 8, 8.3%), angiosarcoma (AS; n = 6, 6.3%), Ewing sarcoma (ES; n = 5, 5.2%), Kaposi sarcoma (n = 4, 4.2%), and various other subtypes of STS (n = 14, 14.6%). Following the proper diagnosis of STS, the patients received oncological treatment, specified in Supplementary Material S2. Among 80 patients with known outcomes, 52 were described as having no evidence of disease (NED), 13 as alive with disease (AWD), and 15 as died of disease (DOD). The median follow-up time (reported in 64 patients) was 16.5 months (range: 0–144 months; IQR: 7.75–29 months).

### 4.1 Infantile fibrosarcoma

Among all identified case reports, 23 pertained to IFS. Of these, 11 involved male patients and seven involved female patients; in five cases, sex was unspecified. Almost all tumors (22/23; 95.7%) were congenital, and one developed at the age of 6 months. Clinical presentation was described in 22 patients, all of whom presented with a swelling or a mass. The majority of the tumors (n = 16; 69.6%) were located on the extremities, followed by the trunk (n = 4, 17.4%), head and neck (n = 2; 8.7%), and a single case involving the head, neck, and trunk (n = 1; 4.3%). Additional symptoms included bleeding in five cases, ulceration in four cases, and pain in one case. The mean maximum tumor size at initial presentation was 45 mm (based on 10 cases), increasing to 70 mm at STS diagnosis (14 cases). In all four cases where both measurements were available, the tumor progressed, with the median maximum size increasing from 30 mm to 72.5 mm.

Misdiagnosis was solely clinical in 12 (52.2%) cases and both clinical and radiological in 11 (47.8%) cases. The most common initial misdiagnosis was hemangioma (15 cases, including seven congenital hemangiomas) and lymphatic or lympho-venous malformation (seven cases). One lesion was misdiagnosed as an unspecified vascular anomaly (hemangioma or lymphatic malformation). Diagnostic delay ranged from 3 weeks to 21 months, with a median of 2 months. Fourteen patients received treatment for the misdiagnosed benign VA, including local therapies (surgery, laser therapy, sclerotherapy, topical timolol, or thrombin) in 11 children and medical therapies (GCS, propranolol, or antibiotics) in six. Despite initial misdiagnoses, the prognosis remained good, with only one reported death due to malignancy.

### 4.2 Alveolar soft-part sarcoma

The literature review revealed 22 cases of ASPS misdiagnosed as benign VA—13 female and 9 male patients. Additionally, a patient from our institution described above was included in the analysis, yielding 23 cases. The majority (15/23, 65.2%) of the patients were children, with a median age of 14 years (range: newborn to 52 years). The lesions were most commonly located within the head and neck (n = 12; 52.2%), followed by the limbs (n = 10; 43.5%) and trunk (n = 1; 4.3%). The most frequently reported symptoms at initial presentation were swelling or a palpable mass (n = 17; 73.9%) and pain (n = 4; 17.4%). Other mentioned symptoms included discomfort, visible collateral vessels, bleeding, and functional impairments such as problems in swallowing, chewing, and articulation, along with proptosis, exophthalmos, strabismus, and anemia. The misdiagnoses were both clinical and radiological in nine cases (39.1%), clinical only in five cases (21.7%), and radiological only in three cases (13.0%) and included hemangioma in 17 cases (73.9%), followed by AVM or other high-flow vascular malformations in five cases (21.8%), and unspecified vascular malformation in one case (4.3%).

Diagnostic delay was reported in 13 patients and ranged from 2 months to over 10 years, with a median of 12 months. Seventeen patients received treatment for the presumed AVM, which included mainly local therapies (surgery in 14; embolization or chemoembolization in 7; laser therapy, cryotherapy, and intralesional GCS in 1 each). One patient received systemic treatment with propranolol and sirolimus. Prognosis in this group of patients varied, with nine patients reported as NED, seven as AWD, and two as DOD at the last follow-up.

### 4.3 Rhabdomyosarcoma

There were 13 cases of RMS misdiagnosed as benign VA. Histopathological subtypes included six embryonal, two alveolar, and five unspecified cases. Consistent with the typical age distribution of this malignancy, all patients were children. However, nine of them were diagnosed in the first year of life (median age: 4 months; range: newborn–10 years). The most frequently affected anatomical region was the head and neck (eight patients), followed by the extremities (five cases, of which three were localized within the hand). The clinical manifestation was described in 11 patients and predominantly included a mass or swelling (n = 10, 76.9%). One child with widely spread alveolar RMS also experienced pruritus and acholic stools, whereas another patient with an orbital tumor presented proptosis. In another patient with an orbital tumor, the lesion was not externally visible; however, symptoms included proptosis, lateral displacement of the eye, and restricted ocular motility. Misdiagnoses were based on clinical (n = 3, 23.1%), radiological (n = 2; 15.4%), or both clinical and radiological (n = 8, 61.5%) assessments. The initial incorrect diagnoses included hemangioma in seven children (of which one was specified as infantile and one as congenital hemangioma), lymphatic malformation or “lymphangioma” in four cases, and a nonspecific vascular malformation in one. In one child, multiple diagnoses of benign VAs were proposed.

Six children received treatment for the initially diagnosed benign VA, including surgery (n = 2), embolization (n = 1), intralesional GCS (n = 1), and medical therapies with propranolol and GCS (n = 3). The outcomes following diagnosis of RMS varied; three patients died of the disease (two within 2 months since diagnosis), one remained AWD at a 6-month follow-up, and five were reported as NED. Four children were lost to follow-up.

### 4.4 Dermatofibrosarcoma protuberans

There were eight patients with DFSP misdiagnosed as benign VA: five female and three male patients. Among them, one had a mixed histopathology with giant cell fibroblastoma (12). Two patients had the pigmented variant of DFSP (Bednar tumor) (13,14). All initially presented in childhood (median age: 8 months, range newborn–8 years), including two congenital tumors. Five DFSPs were localized on the extremities, and three in the trunk. In four patients, the lesion was described as a plaque, whereas three had a more tuberous swelling/mass. In one child, no description of the lesion’s characteristics was provided. Misdiagnoses were mainly clinical (n = 5) or both clinical and radiological (n = 3) and included a wide spectrum of benign VAs, including hemangiomas and vascular malformations. The diagnostic delay ranged from 6 months to 30 years (median: 5 years). Despite misdiagnoses, all seven patients with known follow-ups are alive with NED, with a median follow-up of 20 months.

### 4.5 Angiosarcoma

We identified six patients with AS who were initially misdiagnosed with benign VA. In four of them, the lesion developed during infancy, including one congenital case, whereas the remaining two patients were diagnosed at 59 and 67 years of age, respectively. The most commonly affected anatomical region was the head and neck, with four cases, including two involving the nose; however, it remains uncertain whether these nasal presentations were primary cutaneous AS or represented metastatic lesions known as “clown nose sign” (15). One tumor affected the abdominal wall and one the lower extremity. The clinical presentation was described in five patients and included a mass or swelling (n = 3; 50.0%), which in one orbital lesion was also associated with proptosis, or a papule (n = 2; 33.3%). In five cases, the misdiagnosis was based on both clinical and radiological assessment and was based solely on clinical evaluation in one case. The initial incorrect diagnoses included hemangioma (four cases, with two specified as lobulated capillary hemangioma/pyogenic granuloma and one as infantile hemangioma), lymphatic malformation (one case), and unspecified benign VA (one case). In this patient group, four concomitant pathological misdiagnoses were also identified (16–18); however, in case of one infant, it was uncertain whether the misdiagnosis resulted from nonrepresentative biopsy material or was an example of a rare and not fully proven phenomenon of malignant transformation of infantile hemangioma to AS (19). All patients received treatment for presumed benign VA, including surgery (n = 4; 67.7%), laser therapy (n = 2; 33.3%), and various systemic treatments, such as GCS, propranolol, sirolimus, interferon alpha-2a, and chemotherapy (n = 3; 50%). Following the proper diagnosis of AS, five patients received additional oncological treatment, as detailed in Supplementary Material S2. The prognosis was poor, with five patients reported as DOD and one as NED but later lost to follow-up.

### 4.6 Ewing sarcoma

The analyzed cohort included five patients (three male and two female patients) with soft-tissue ES misdiagnosed as benign VA. All presented in prepubertal age (median 5 years, range 0–11 years). In one patient, the tumor was first visualized in prenatal ultrasound and exhibited restricted diffusion in both prenatal and postnatal MRIs. Nevertheless, after birth, propranolol was started without performing a tumor biopsy due to the features of the lesion in physical examination, suggesting infantile hemangioma.

Four out of five tumors were localized on the extremities (among them, three affected the hand), and one involved the trunk. All patients presented with a swelling/mass, which was accompanied by pain in two. The misdiagnosis was clinical in two and both clinical and radiological in three children and included hemangioma in three and vascular malformation in two patients. One patient each received antibiotics and propranolol for the treatment of misdiagnosed benign VA, and four patients were ultimately qualified for surgery, which led to the final diagnosis of ES. The diagnostic delay ranged from 4 months to 4 years. After ES diagnosis, all patients received multimodal oncological treatment. At the time of publication, four out of five patients remained NED, with a median follow-up of 1 year (two were still on treatment), and one patient died due to ES progression shortly after diagnosis.

### 4.7 Kaposi sarcoma

Four cases of Kaposi sarcoma were identified, all in male adult patients, with a median age at diagnosis of 54 years (range: 37–88 years). Three of them had confirmed human immunodeficiency virus (HIV) infection. Two tumors involved the head and neck, whereas two were located on the extremities. Initial misdiagnoses were clinical in two cases and both clinical and radiological in another two. They included benign vascular tumors and vascular malformations in two patients each. Only one patient received surgical treatment for the presumed benign VA. The diagnostic delay was provided in three patients and ranged from 1 month to 1 year. The outcome was documented in two cases, both reported as NED.

### 4.8 Other diagnoses

There were 13 patients diagnosed with other types of STS. This heterogeneous group included two cases of infantile myofibromatosis and single cases of BCOR-rearranged undifferentiated sarcoma, leiomyosarcoma, low-grade soft-tissue sarcoma, synovial sarcoma, low-grade spindle cell sarcoma, “poorly differentiated round and spindle cell sarcoma,” “infantile rhabdomyofibrosarcoma,” malignant rhabdoid tumor, hemangiopericytoma, histiocytoma, and malignant peripheral nerve sheath tumor. These tumors occurred mainly in infants (n = 9; 69.2%), with one child diagnosed at the age of 7 years and three patients diagnosed in adulthood. Misdiagnoses included vascular malformation in seven cases (further specified as AVM in three and lymphatic malformation in one) and hemangioma in six cases (specified as infantile hemangioma, congenital hemangioma, “capillary hemangioma,” and disseminated hemangiomatosis, one each). The median diagnostic delay was 5.5 months (range: 1 week to 5 years). Four patients received treatment for the presumed benign VA—surgery (n = 2; 15.4%), embolization (1 = 1; 7.7%), and GCS (1 = 1; 7.7%). Due to the heterogeneity of this group, oncological treatment methods and outcomes varied substantially (see Supplementary Material S2).

## 5 Discussion

In this paper, we provide a systematic review of 96 patients with STSs misdiagnosed as benign VAs based on clinical or radiological features. Our intent was to analyze the causes responsible for the delayed and improper diagnoses. We have identified several factors that may contribute to the considerable risk of misdiagnosis in patients suspected for hemangioma or vascular malformation. The median diagnostic delay in our cohort was 5 months, ranging from 1 week to 30 years. Due to the limited information provided in the articles, we could not reliably divide the diagnostic delay into “patient-related” and “healthcare-related”; however, in many cases, multiple contacts with healthcare providers preceded obtaining the proper diagnosis.

### 5.1 Selected patterns of misdiagnoses can be distinguished

In our systematic review cohort, several “clusters” of misdiagnosed cases could be identified, for example, IFS or RMS misdiagnosed as infantile or congenital hemangiomas or ASPS misdiagnosed as AVM. These findings may result from a clinical and radiological resemblance between some types of STS and benign VAs. For example, IFS and congenital or infantile hemangiomas share a similar age at presentation and, in many cases, similar clinical features (20,21). In addition, RMS may present in infancy as a fast-growing soft tissue lesion, mimicking infantile hemangioma (22). All these entities may present with bluish hue and extensive vascularization (23). Moreover, rapid growth of the lesion, ulceration, and bleeding, which in older child or adult would immediately raise strong suspicion of malignancy, may, in the first weeks or months of life, be attributed to the natural history of infantile hemangioma, leading to delayed diagnosis (20,24,25). Infantile fibrosarcoma and RMS may also present as multiseptated, cystic masses, resembling a lymphatic malformation (26–29). Alveolar soft-part sarcoma, which is a highly vascularized STS, may, in turn, show striking similarities to AVM, including localization (head and neck in children and deep extremity muscles in adults), symptoms (pulsatile mass with an audible bruit), and radiological features on angiography and MRI (tortuous feeder vessels and flow voids) (30–32). Some authors have suggested the role of 18FDG-PET-CT in discriminating between ASPS and benign VAs (33). Some consistent radiological features of ASPS, not typically present in AVM, include high signal intensity in MRI T1-weighted sequences, delayed contrast washout, and the presence of a solid mass component, accompanied sometimes by central necrosis (30,34,35). However, in our patient, the image of the lesion in the first MRI (Figure 3) was ambiguous, probably due to its relatively small size (below 40 mm). Although the mass had several features typical for ASPS, as described by Crombe et al. (30), including intra- and peritumoral flow voids, no tail sign, and no fibrotic component, it lacked many other characteristics of ASPS—it was not located very deeply, was not hyperintense in the T1-weighted sequences, had no central necrosis area, and did not exhibit an infiltrative growth pattern. The exact number of flow voids was difficult to assess. In the analyzed cohort, as many as seven patients with ASPS (including our patient) underwent embolization procedures for presumed benign VA, which could have contributed not only to delayed proper ASPS treatment but also to the dissemination of the cancer cells and ultimately worse outcome. Another example is pyogenic granuloma, which is a fast-growing, bleeding lesion and may show clinical similarities with Kaposi sarcoma or skin AS (16,36). Only a deep knowledge of nuances differentiating clinical and radiological pictures of benign VAs and STSs may allow clinicians to avoid misdiagnosis and improper treatment.

Another commonly reported reason for misdiagnosis of several types of STSs as benign VAs is relatively indolent clinical behavior of these sarcomas. For example, DFSP, despite being a locally aggressive cutaneous STS, may grow slowly and remain asymptomatic for many years (37). These features are shared with vascular malformations. A delayed diagnosis of DFSP may result in the need for more extensive surgery and reconstructive procedures or adjuvant radiotherapy, increasing the patient’s morbidity (38). Similarly, ASPS may initially develop with an indolent phase lasting even for a few years, which, together with radiological features, may favor a diagnosis of vascular malformation (32). In case of ASPS, delayed diagnosis is especially dangerous as this STS is curable only in the early, localized stage. This was the case of the patient from our institution, in whom the head-and-neck ASPS developed indolently for the first 7 years, followed by rapid growth and progression during puberty, and massive dissemination to multiple organs after several embolization procedures.

### 5.2 The knowledge of the classification, biology, and clinical characteristics of benign VAs among physicians is insufficient

In several analyzed papers, the authors used improper and misleading names of benign VAs, which are not supported by the ISSVA classification, such as “cavernous hemangioma,” “lymphangioma,” “tuberous angioma,” or “capillary hemangioma” (20,29,38–40). Moreover, in many publications, the initial diagnosis of benign VA was inconsistent with the provided description of the lesion (e.g., “infantile hemangioma” used for lesions present and fully formed at birth, or “hemangioma” used for tumors first manifesting beyond infancy) (33,41–46). In some cases of IFS or RMS misdiagnosed as hemangiomas, it is apparent that the clinical history fitted neither congenital nor infantile hemangioma (e.g., lesion fully developed at birth, but exhibiting further rapid growth during first months of life) (47). These findings are consistent with the studies of Hassanein et al. and Steele et al., who reported that in the majority of publications, the denominations used to describe benign VAs were incorrect, and patients whose lesions were misnamed were more likely to receive inappropriate treatment (48,49). This is particularly concerning as benign VAs are common diseases (e.g., infantile hemangiomas affect up to 5% of full-term infants). Similar situations could also be found in the publications reviewed in our study—for example, the neonate reported by Yang et al. received propranolol for a suspected infantile hemangioma despite being born with a fully formed lesion. The biopsy of the mass was not performed even though the MRI features of the lesion strongly suggested malignancy (50). In another patient with mixed DFSP/giant-cell fibroblastoma histology, the deep-seated arm lesion was detected at 6 months of age and grew rapidly until the age of 1 year. The child was initially diagnosed with infantile hemangioma based on clinical and radiological features (12) despite the fact that infantile hemangioma very rarely presents as late as at 6 months of age. According to literature data, any lesion resembling infantile hemangioma, but with first symptoms developing after 3–4 months of age, should be presumed not to be infantile hemangioma and requires diagnostic workup (51).

### 5.3 There is insufficient awareness of rare types of STSs, whose clinical and radiological characteristics may resemble benign VAs

Pediatricians and other specialists are not sufficiently aware of the fact that some types of STS may present with clinical features very similar to benign VAs. For example, DFSP is very rare in children and much less frequent than benign VAs. This may be the reason why in the analyzed cohort, all misdiagnosed patients with DFSP were children. A comparable situation was observed in the case of AS, a malignancy typically presenting in individuals over 70 years of age (52). This epidemiological profile likely contributed to the omission of AS in the differential diagnosis of the four analyzed infant cases, three of whom eventually died due to disease progression.

Similarly, ASPS is a rare type of STS, especially in children, and some clinicians and radiologists may not be aware that it should be included in the differential diagnosis of deep-seated AVM.

### 5.4 In case of any ambiguity, clinical and radiological assessment of benign VAs should be complemented by histopathological analysis

Although clinical examination and imaging—primarily ultrasound, followed by MRI—serve as fundamental tools for diagnosing benign VAs, they may not always be sufficient for accurate diagnosis. Therefore, in both congenital and acquired lesions with unclear clinical presentation, significant growth dynamics, or unsatisfactory response to the standard treatment, performing a biopsy appears warranted (7). Moreover, in case of unexpected laboratory findings, such as HIV infection, cautious differential diagnosis is necessary (46,53). It is important to note that in some cases, treatment administered for benign VAs may produce a transient effect in STS, further obscuring the clinical picture. Such a situation occurred in the patient from our institution, in whom embolization procedures performed to treat the presumed AVM, led to temporary stabilization of ASPS by reducing tumor vascularization. Similar cases have been reported in the literature, for example, a transient response of IFS and RMS to oral or intralesional GCS, probably resulting from the reduction in tissue edema surrounding the tumor (24,54,55).

If indicated, the biopsy should not be deferred due to the risk of hemorrhage. Patients at an increased risk of bleeding from the benign VA (e.g., those with AVMs) should receive hematological support during the pre- and postoperative periods. It is important to emphasize that the histopathological differential diagnosis between benign VAs and STS is sometimes difficult—in our cohort, at least eight misdiagnoses were, at some point, also pathological. Therefore, it is essential to obtain an optimal representative sample, rather than only the surrounding reactive tissue or vessels supplying the lesion (27). The specimen should be assessed independently by two experienced pathologists to enhance diagnostic accuracy.

### 5.5 Patients with an atypical clinical course of suspected benign VAs should be consulted by multidisciplinary vascular anomaly team to increase the chance for an early proper diagnosis and treatment

Benign VAs are relatively common (56). Many of them, including uncomplicated infantile hemangiomas or small and stable vascular malformations, may be safely managed in outpatient settings, by primary care physicians, pediatricians, or surgeons, depending on the clinical situation and local practice. However, patients who present with an atypical course of benign VA should be immediately referred to a specialist experienced in the management of VAs and, optimally, be consulted by the multidisciplinary vascular anomaly team. In some cases, even initial evaluation of the referral documents by experienced physicians may raise suspicion of misdiagnosis (7).

In complicated cases, making the proper diagnosis often requires collaboration among internal medicine physicians or pediatricians, surgeons, radiologists, interventional radiologists, pathologists, and oncologists. This can be achieved by establishing multidisciplinary vascular boards, which can integrate different perspectives and resolve ambiguities, resulting in improved diagnostic accuracy and treatment outcomes.

### 5.6 Limitations of the study

This study is prone to reporting bias, as misdiagnosed cases are more likely to be published. This may lead to an overestimation of the prevalence of diagnostic errors.

## 6 Conclusions

To avoid misdiagnoses, patients with benign VAs should be diagnosed and treated by specialists familiar with the current ISSVA classification and natural history of hemangiomas and vascular malformations. In patients with benign VAs diagnosed based on clinical and radiological features only, all history, physical examinations, and imaging findings should be unequivocally consistent with the suspected diagnosis. In case of any ambiguities, a patient should be immediately referred to the tertiary center for the multidisciplinary vascular team consultation. The lesion biopsy should be performed in doubtful or atypical cases to obtain histopathological examination and, if necessary, molecular studies.

## Data Availability

The original contributions presented in the study are included in the article/Supplementary Material; further inquiries can be directed to the corresponding author.
