# Supplementary material for: Diagnostic pitfalls: soft-tissue sarcomas initially misdiagnosed as benign vascular anomalies—a case report and systematic review
Source: Oncol Rev. 2025 Dec 11;19:1681090. doi: 10.3389/or.2025.1681090 (PMC12738830; doi:10.3389/or.2025.1681090)
Supplement: Supplementary file 1 [file Supplementaryfile1.pdf]

1. Inclusion criteria

- all types of studies (including case-reports and case series)
- patients with histopathological diagnosis of STS, clinically and/or radiologically diagnosed as benign vascular anomaly (defined as a vascular malformation or a benign vascular tumor, according to International Society for the Study of Vascular Anomalies [ISSVA] classification)

2. Exclusion criteria

- patients with clinical and radiological suspicion of STS
- patients with histopathological diagnosis of a benign VA

3. Search Strategy:

a) PubMED/MEDLINE (MeSH terms)

sarcoma	AND	vascular malformations OR hemangioma	AND	diagnostic errors

b) PubMED/MEDLINE (title, abstract)

sarcoma OR		vascular malformation OR		misdiagnosis OR
rhabdomyosarcoma OR		arteriovenous malformation OR		misdiagnosed OR
leiomyosarcoma OR		venous malformation OR		mistake OR
angiosarcoma OR	AND	lymphatic malformation OR	AND	mistaken OR
liposarcoma OR		capillary malformation OR		pitfall OR
dermatofibrosarcoma OR		hemangioma OR		differential OR
fibrosarcoma		vascular anomaly		mimicking OR
				mimicker

c) Web of Science (topic)

sarcoma	AND	vascular malformation	AND	misdiagnosis

OR		OR		OR
rhabdomyosarcoma OR		arteriovenous malformation OR		misdiagnosed OR
leiomyosarcoma OR		venous malformation OR		mistake OR
angiosarcoma OR		lymphatic malformation OR		mistaken OR
liposarcoma OR		capillary malformation OR		pitfall OR
dermatofibrosarcoma OR		hemangioma OR		differential OR
fibrosarcoma		vascular anomaly		mimicking OR
				mimicker

d) Scopus (title, abstract, keywords)

sarcoma OR	AND	vascular malformation OR	AND	misdiagnosis OR
rhabdomyosarcoma OR		arteriovenous malformation OR		misdiagnosed OR
leiomyosarcoma OR		venous malformation OR		mistake OR
angiosarcoma OR		lymphatic malformation OR		mistaken OR

liposarcoma OR		capillary malformation OR		pitfall OR
dermatofibrosarcoma OR		hemangioma OR		differential OR
fibrosarcoma		vascular anomaly		mimicking OR
				mimicker

f) EMBASE (title, abstract, keywords)

sarcoma OR		vascular malformation OR		misdiagnosis OR
rhabdomyosarcoma OR		arteriovenous malformation OR		misdiagnosed OR
leiomyosarcoma OR		venous malformation OR		mistake OR
angiosarcoma OR	AND	lymphatic malformation OR	AND	mistaken OR
liposarcoma OR		capillary malformation OR		pitfall OR
dermatofibrosarcoma OR		hemangioma OR		differential OR
fibrosarcoma		vascular anomaly		mimicking OR
				mimicker

g) EMBASE (Emtree)

sarcoma	AND	arteriovenous malformation OR	AND	diagnostic error

		lymphangioma<br><br>OR<br><br>benign vascular tumor		

h) Cochrane Central Register of Controlled Trials (CENTRAL) - trials

sarcoma<br>OR	AND	vascular malformation<br>OR	AND	misdiagnosis<br>OR
rhabdomyosarcoma<br>OR		arteriovenous malformation<br>OR		misdiagnosed<br>OR
leiomyosarcoma<br>OR		venous malformation<br>OR		mistake<br>OR
angiosarcoma<br>OR		lymphatic malformation<br>OR		mistaken<br>OR
liposarcoma<br>OR		capillary malformation<br>OR		pitfall<br>OR
dermatofibrosarcoma<br>OR		hemangioma<br>OR		differential<br>OR
fibrosarcoma		vascular anomaly		mimicking<br>OR<br>mimicker
